# Novel sites for pacemaker lead implantation and different routes for their extraction

**DOI:** 10.1007/s12471-022-01684-w

**Published:** 2022-04-20

**Authors:** J. R. de Groot

**Affiliations:** 1 Department of Cardiology, Heart Centre, Amsterdam University Medical Centres, University of Amsterdam, Amsterdam, The Netherlands; 2 Amsterdam Cardiovascular Sciences, Heart failure & arrhythmias, Amsterdam, The Netherlands

Permanent cardiac pacemakers have been standard therapy for bradycardia and conduction disturbances for more than six decades. Since the first pacemaker implantation in 1958, on-demand dual chamber pacing and algorithms to optimise atrioventricular synchrony and promote intrinsic conduction have been developed, cardiac resynchronisation has been introduced, and completely leadless systems have been implanted in the right ventricle—most often in the right ventricular apex. However, chronic right ventricular apical pacing results in cardiac dyssynchrony, which may lead to pacing-induced heart failure, particularly in patients with diminished left ventricular function [1]. Although algorithms to reduce right ventricular apical pacing are available and alternative pacing sites, including right interventricular septum and right ventricular outflow tract, have been proposed, clinical evidence of their superiority over left ventricular apical pacing in patients with preserved left ventricular function has not been established [2].

This has led to the introduction of conduction system pacing: pacing from the His bundle or left bundle branch (LBB) to maintain the normal ventricular activation sequence and prevent pacing-induced heart failure. In this issue of the* Netherlands Heart Journal*, which is dedicated to electrophysiology in Dutch clinical practice, Heckman and colleagues describe their investigation of the feasibility and the learning curve of LBB area (LBBA) pacing [3]. LBBA capture was diagnosed when decreasing pacing output resulted in transition from non-selective LBB pacing, via selective LBB pacing, to myocardial-only capture and a similar interval between LBB potential and peak R wave in lead V6 as between pacing stimulus and peak R wave in V6 (ventricular activation time). In 80 consecutive patients, a successful implant was achieved in 96%. LBBA capture, however, was achieved in 54/80 patients (68%) and was associated with a significantly shorter paced QRS duration than without LBB capture. The authors conclude that LBBA is associated with ventricular synchrony with QRS durations close to intrinsic (narrow QRS) rhythms [3].

Subsequently, Rademakers and colleagues report on their first experience with LBB pacing [4]. They describe 100 consecutive patients, of whom 57 had left ventricular ejection fractions < 50%. Successful LBB pacing was achieved in 83% of patients, but the success rate tended to be lower in patients with an indication for cardiac resynchronisation. Similar to the former study, ventricular activation time was defined as the time interval from the pacing spike to the R wave in V5-V6. LBB capture was confirmed when the paced QRS showed right bundle branch block morphology in combination with ventricular activation time < 90 ms.

One of the major disadvantages of (transvenous) pacing, including LBB pacing, is the potential need for extraction of one or more pacemaker leads during follow-up for various reasons. Bracke et al. therefore report probably the largest series of lead extractions in the Netherlands [5]. Between 1997 and 2019, 1725 leads were extracted in 775 patients using subclavian laser sheath extraction, a femoral approach or rotating mechanical sheaths. All three extraction strategies were similarly effective, but more major complications (mainly superior caval vein lesions and intrapericardial tears, but also mortality) occurred in the laser sheath group (8.4%) than in the femoral approach group (0.5%) or the rotating mechanical sheath group (1.2%). Of the 24 patients with major complications, 21 were immediately operated, underscoring the need for cardiothoracic surgical backup when performing these types of procedures. The authors recommend a hybrid approach for lead extraction, with a femoral approach for leads in the atrium and/or coronary sinus combined with a mechanical rotating sheath introduced from the subclavian vein for right ventricular leads [5].

This Electrophysiology issue is complemented with two papers on atrial fibrillation ablation. Van Deutekom and colleagues report their series of cryoballoon ablation for atrial fibrillation, with emphasis on heart rate increase and inappropriate sinus tachycardia [6]. From their experience of more than 600 cases, they report on 169 patients (other patients were mainly excluded because of missing Holter follow-up recordings or presence of atrial arrhythmias on pre- or postprocedural Holter monitoring). The authors show that abnormal heart rate response after cryoballoon pulmonary vein isolation is rare and for the largest part reversible during follow up. Only 4% of the patients conformed to the criteria for inappropriate sinus tachycardia.

Liebregts et al. describe their first experience with the AcQMap system to ablate complex atrial arrhythmias using multi-electrode non-contact mapping in 21 patients [7]. At 12 months, 4 patients treated for persistent atrial fibrillation (29%) and 4 patients treated for atypical atrial flutter (57%) remained in sinus rhythm. This modest result may be related to low patient numbers and the potential learning curve of the system, but it is likely to resemble, in part, the advanced atrial pathology.

Together, these contributions to this special issue of the* Netherlands Heart Journal* provide us with insight and understanding of new techniques, but more importantly, they underscore the continuous innovation in our field.
